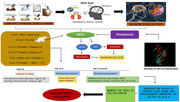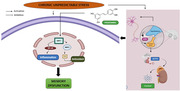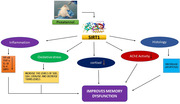# Neuroprotective role of Piceatannol in Chronic Unpredictable Stress induced memory dysfunction

**DOI:** 10.1002/alz.087954

**Published:** 2025-01-09

**Authors:** Amarjot Kaur Grewal, Parneet kaur Dhaliwal, Pragati Silakari, Amit kumar, Heena Khan, Thakur Gurjeet Singh

**Affiliations:** ^1^ Chitkara College of Pharmacy, Chitkara University, Rajpura, Punjab India

## Abstract

**Background:**

Various studies have evidenced the neuroprotective role of SIRT1 activator. However, whether SIRT1 activator, Piceatannol pharmacological treatment is protective in chronic unpredictable stress induced memory dysfunction remains unknown. Therefore, this study design included testing the hypothesis that Piceatannol administered in chronic unpredictable stress induced memory dysfunction mice shows protective effects, explores & probes underlying the activation of SIRT1 pathway.

**Method:**

Swiss albino male mice were subjected to chronic unpredictable stress which further resulted in memory dysfunction. The levels of cortisol, biochemical parameters, inflammatory mediators, and AChE activity were examined. For memory assessment, mice were subjected to morris water maze (MWM) and passive avoidance test. Histological changes were assessed using HE staining.

**Result:**

Piceatannol (10 & 20 mg/kg; *oral*) dose dependently reduced chronic unpredictable stress induced memory dysfunction’s deleterious effects on biochemical parameters of oxidative stress (SOD, GSH, Catalase, TBARS), inflammatory parameters (IL‐6, IL‐1β, NF‐κB, TNF‐α, MPO), TSTQ, Transfer latency time, cortisol levels, AChE activity and histopathological changes, according to the findings. These neuroprotective effects of Piceatannol were significantly abolished by pre‐treatment with Sirtinol (10 mg/kg; *i.p*), a SIRT1 inhibitor. Current study concluded that induced neuroprotective benefits of Piceatannol (10 & 20 mg/kg; *oral*) in all probability, maybe mediated through SIRT1 activation. Hence, its neuroprotective effects can be further explored in clinical settings.

**Conclusion:**

From the evaluation of the results of numerous parameters, we draw the conclusion that the neuroprotective effects of Piceatannol in chronic unpredictable stress induced memory dysfunction may have been caused by SIRT1 pathway activation.